# A Novel Approach to the Reconstruction of a Large Surgical Defect in the Cheek

**DOI:** 10.3390/jcm12247750

**Published:** 2023-12-18

**Authors:** Verónica Ruiz Salas

**Affiliations:** Dermatology Department, Universitat Autònoma de Barcelona, Sant Pau Campus Salut Barcelona, C/Mas Casanovas 90, 08041 Barcelona, Spain; v.ruizsalas@hotmail.com; Tel.: +34-935567007; Fax: +34-9-35567008

**Keywords:** larger cheek defects, double transposition flap, basal cell carcinoma, cheek reconstruction, Mohs surgery

## Abstract

Background: Large lateral cheek defects can be challenging to reconstruct. Several approaches to reconstruction of these defects have been reported. In the case presented here, we describe an alternative reconstruction method for this type of surgical defect. Detailed Case Description: We present one patient with a large basal cell carcinoma on his lateral left cheek who underwent a complete tumor removal by Mohs surgery and was left with a defect 6 × 6 cm in size. This large defect was closed by performing a double transposition flap under local anesthesia. Results: Both flaps survived with no loss. The immediate and long-term outcomes were satisfactory, preserving functionality with good cosmetic results. Conclusions: Cheek defect reconstruction with the double transposition flap is simple and reliable, with good aesthetic and functional outcomes. It may be considered as an alternative reconstructive method for this type of defect, in an appropriate context.

## 1. Introduction

The most common malignant tumors that occur on the face are basal cell carcinoma, squamous cell carcinoma, and melanoma. The standard treatment approach for these malignant tumors involves surgical resection. Mohs surgery, if available, is an effective surgical technique used for the removal of certain types of skin cancer, particularly basal cell carcinoma. After resecting the tumor from the cheek, complex reconstruction techniques may be necessary to close the resulting defect. In the case presented here, a novel technique was chosen as a reconstruction method for a large defect in this area.

## 2. Detailed Case Description

### 2.1. Case Description

An 81-year-old Caucasian man presented to our clinic for the treatment of a recurrent infiltrative basal cell carcinoma involving the vast majority of his left cheek. The tumor consisted of an erythematous, partially ulcerated, and poorly defined plaque of 5 × 5 cm located on his left cheek. The tumor had recurred after two previous conventional surgeries. We recommended that he undergo Mohs surgery as the best treatment to achieve a complete removal of the tumor due to the tumor’s large size, history of recurrence, poorly defined clinical margins, and location.

**Figure 1:**
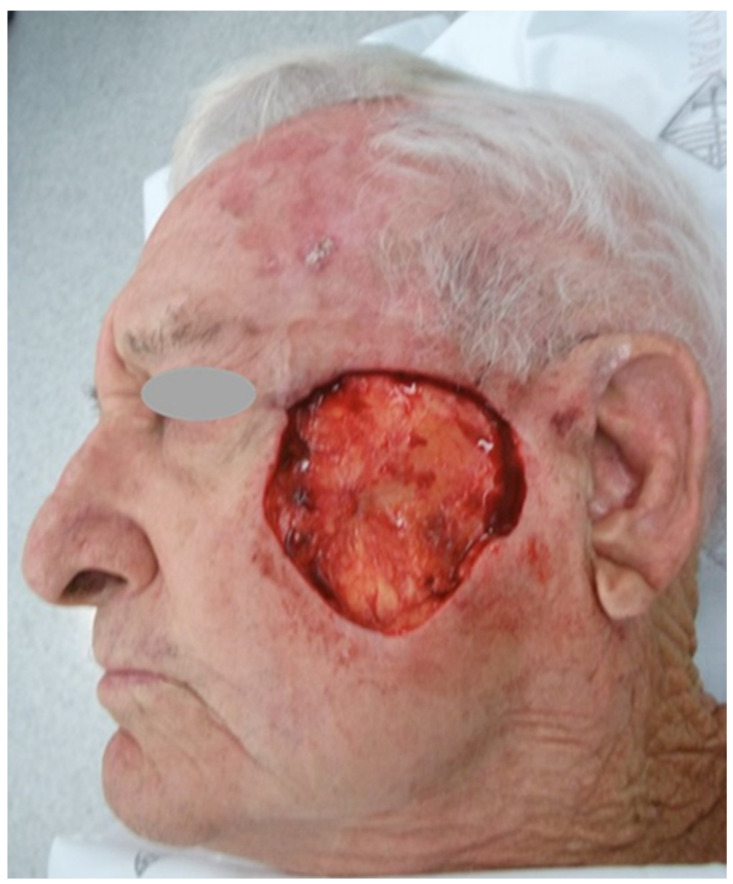
Surgical defect after Mohs surgery.

The patient had been taking direct oral anticoagulants (DOAC) for three years in conjunction with a heart disease. Taking into consideration the size of the tumor and the complexity of the surgery, he was advised to stop the DOAC several days before the surgery in order to reduce the risk of bleeding within the perioperative period. We performed Mohs surgery under local anesthesia, with minimal bleeding, requiring few suture ligatures in order to obtain hemostasis. Clear surgical margins were achieved after two stages of Mohs surgery. The resulting defect measured 6 × 6 cm and was centered primarily on the left lateral cheek (Figure 1).

### 2.2. Technique

Several basic principles must be taken into consideration during this type of surgery. It is necessary to assure complete tumor removal, to restore the anatomical and aesthetic structure of the cheek, to prevent distortion, and to minimize the potential postoperative complications such as alterations of local sensitivity and scar-related complications.

**Figure 2:**
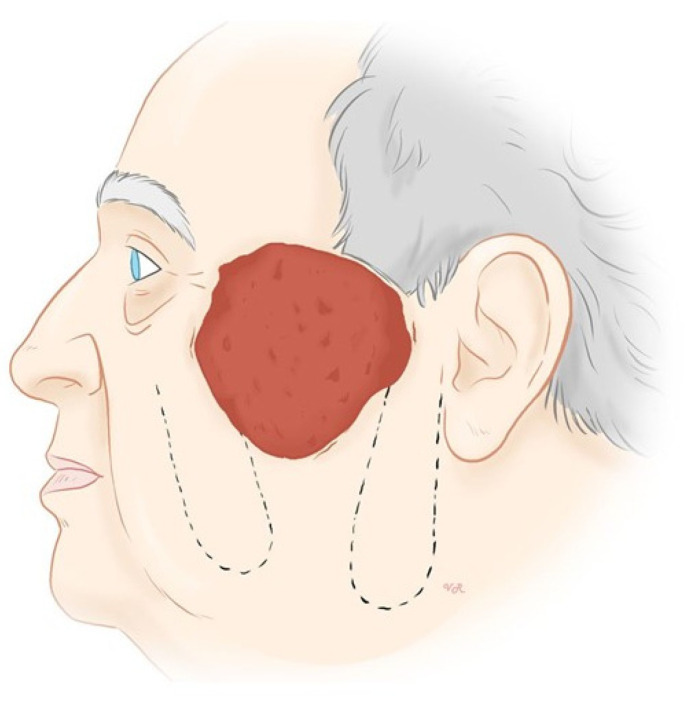
Design of two cutaneous transposition flaps on both sides of the lower half of the defect.

**Figure 3:**
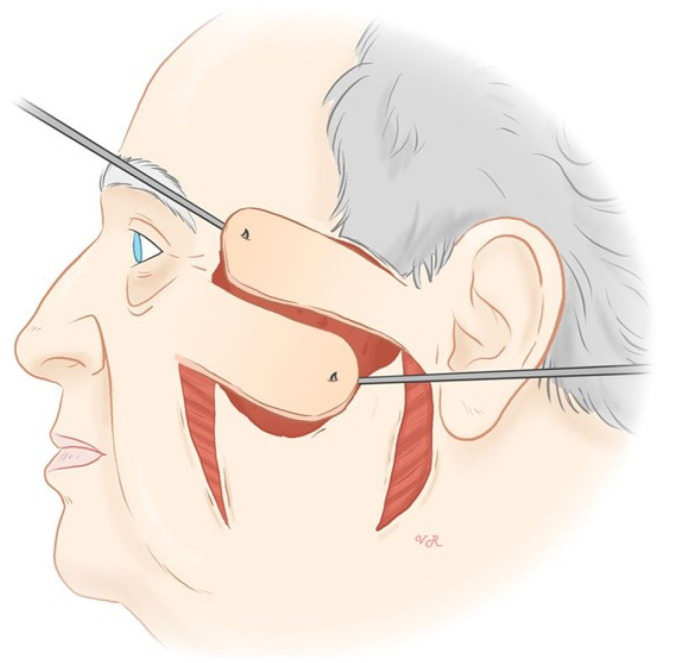
Mobilization of both transposition flaps to cover the surgical defect: the lower right flap was advanced towards the upper half of the defect and the lower left flap was advanced towards the lower half of the defect.

First, the skin was wiped with 2% aqueous solution of chlorhexidine, and the visible tumor was outlined with surgical ink. Anesthesia consisted of local infiltration of a 2% solution of mepivacaine hydrochloride plus a 0.5% solution of bupivacaine hydrochloride. The surgical technique involved the use of two cutaneous transposition flaps (Figure 2). These two flaps were designed on the lower skin in both the left and right sides of the defect. These flaps depend on the rich subcutaneous arterial supply network provided by the transverse facial artery, which is important for nourishing the skin and underlying tissues in the lateral cheek. Both flaps were the same size and were undermined at the subcutaneous plane. After undermining, the lower right flap was advanced towards the upper half of the defect and the lower left flap was advanced towards the lower half of the defect as shown in Figure 3. We first repaired the two secondary defects as a linear closure, allowing better mobilization of both flaps to cover the surgical defect. We used a reabsorbable subcutaneous suture and 5/0 silk. Then, the flaps were sutured, covering the defect, using 4/0 and 5/0 silk. After this, we applied an antibiotic ointment of 2% mupirocin and a petroleum-based gauze dressing. This dressing was covered with a sterile bandage for 1 week, after which the sutures were removed.

### 2.3. Results

**Figure 4:**
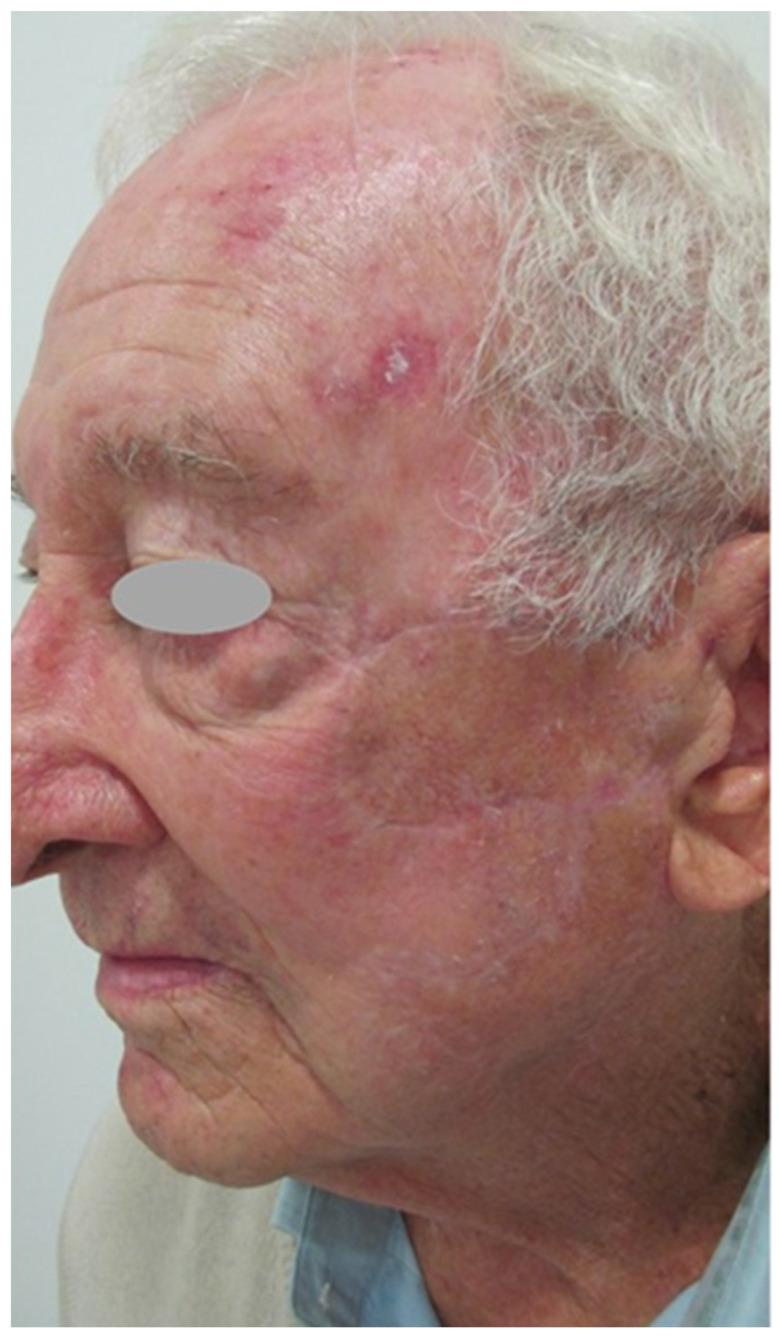
Outcome six months after surgery: Lateral view.

**Figure 5:**
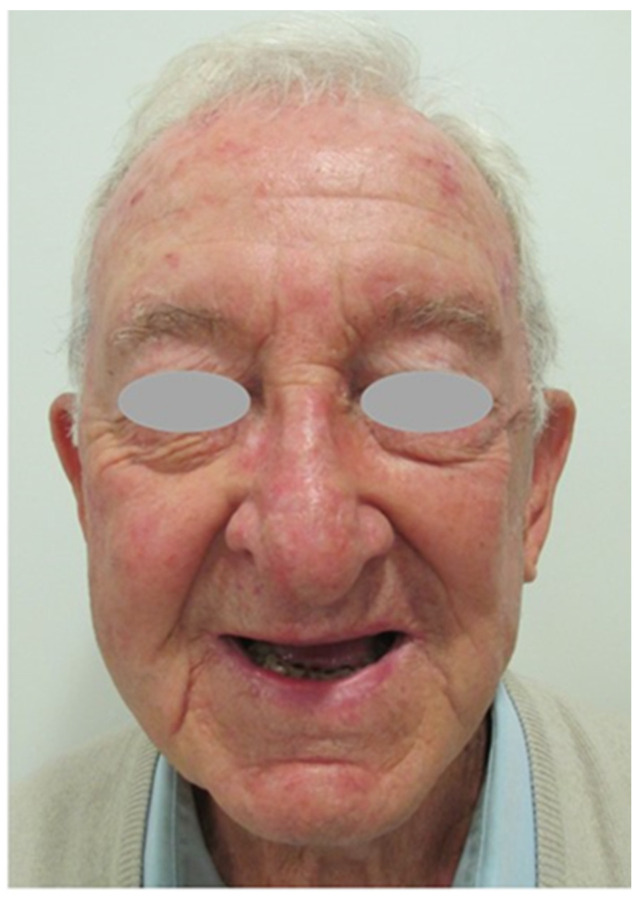
Outcome six months after surgery: Frontal view.

**Figure 6:**
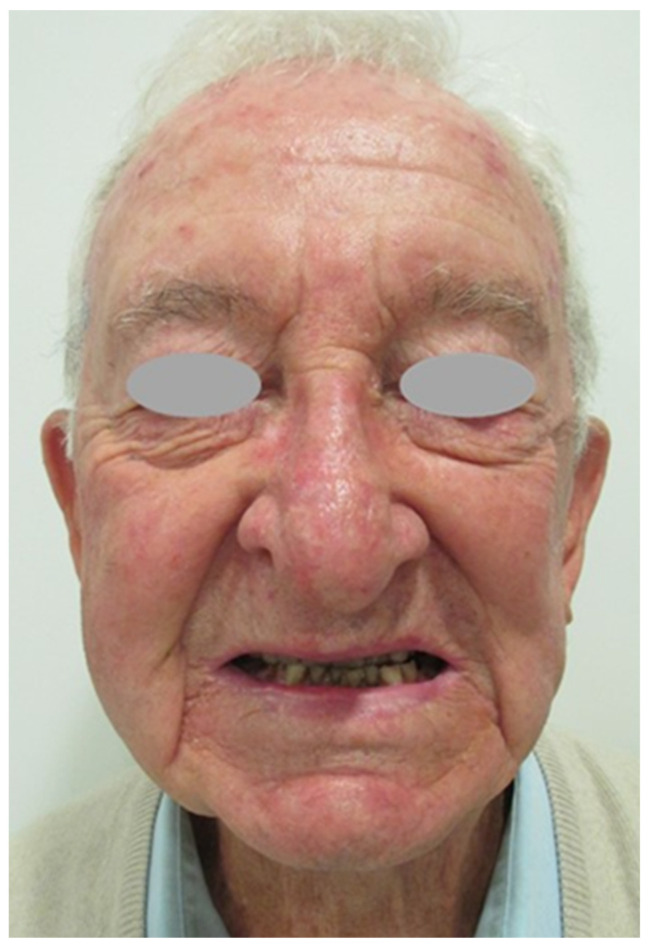
Outcome six months after surgery: Frontal view. There were no nerve injuries during the surgery as shown in this figure. The patient can perform routine facial movements such as smiling.

No local complications were observed after removal of the sterile bandage in this patient. The attached pictures show the scar lines respected the local anatomical integrity, preserving functionality with good cosmetic results. The treatment was successful in preventing the tumor from coming back as no tumor recurrence was observed after six years of follow-up. Figure 4, Figure 5 and Figure 6 show the outcome six months after the surgery.

## 3. Discussion

### 3.1. Anatomy of the Cheek

The cheek or malar zone is the anatomical region of the face which is limited by the eyes, nose, jawline, and ears. The cheeks can participate in eating, talking, and facial expression thanks to this intricate composition.

The cheeks are generally divided into three aesthetic zones: infraorbital, preauricular, and buccomandibular. Some researchers have proposed other forms of subunit classification for the cheek. Weerda et al. divided the cheeks into six anatomical units, namely, the upper, middle, and lateral division of the medial and lateral cheek [1]. Bradley et al. divided the cheeks into medial and lateral (jaw) [2]. Hanks et al. considered it appropriate to categorize the cheek area as follows: medial cheek, perilabial (buccal) cheek, lateral cheek, and zygomatic cheek [3].

The facial artery and the transverse facial artery provide the cheek with its primary blood supply. Both of these arteries are branches of the external carotid artery. The facial artery’s terminal branch, the angular artery, ascends through the lateral contour and supplies blood to the medial cheek border. The zygomatic-orbital artery also supplies blood to the superior border of the cheek. These arteries will continue to develop and create numerous anastomoses to supply collateral blood flow to the cheeks’ structures.

There is an extensive lymphatic drainage system that supplies the cheek region. The submandibular lymph nodes will receive drainage from the medial and inferior cheek regions. The cheek’s superior and lateral regions will drain into the preauricular lymph nodes. They all ultimately drain into the cervical nodes.

Both the facial nerve and the trigeminal nerve supply sensory input to the cheek area. With the exception of the orbicularis oculi and the masseter muscle, the buccal branch of the facial nerve will innervate most of the cheek muscles. The zygomatic branch of the facial nerve supplies the orbicularis oculi muscle with its innervation. The cheeks as well as the rest of the face will receive sensory innervation from the trigeminal nerve. Masseter muscle motor innervation is also provided by the trigeminal nerve.

The area around the cheeks is covered in muscles. In this area of the cheeks, the masseter muscle is the biggest. Although mastication is the masseter’s primary function, it also contributes to the lateral fullness of the cheek [4].

### 3.2. Considerations of Cheek Reconstruction

Large lateral cheek defects can be challenging to reconstruct. When evaluating the best surgical approach for the defect presented here, we need to consider several factors. It is important to assess the size of the defect, underlying structures involved, characteristics of the affected skin (e.g., hair growth), tissue available for reconstruction, and the patient’s individual circumstances. In cheek reconstruction, the main goal is not only to restore the function of the cheek but also to achieve an aesthetic result. For optimal cosmetic results, we need to pay attention to factors such as symmetry, contouring, skin tone matching, and scar management as scars may be noticeable on the cheeks. Therefore, it is recommended to place the closure alignment along the loose skin tension line. Defects can be reconstructed using more than one option, but a specific understanding of the context and patient needs is critical to achieving a successful outcome in each case. The best approach in each may differ depending on the unique needs and conditions of the individual undergoing reconstruction.

In the case presented here, grafts and secondary intention healing are not good choices to reconstruct these defects because they could distort the local anatomy, altering the cutaneous texture.

Secondary intention healing is a process where a wound or surgical defect is left open to heal naturally, without using sutures or closure techniques. It relies on the body’s natural healing mechanisms to gradually close the wound from the base. Secondary intention healing may be an appropriate method for wounds or defects in concave regions of the body such as the inner eye canthus and ear shell because it can help preserve the natural contours and features of these areas. Moreover, in these locations, secondary intention healing may reduce the risk of distortion or scarring that may occur when using other closure methods [5]. In the present case, using secondary intention healing would lead to a higher risk of hypertrophic scarring. This may cause functional issues depending on their location and can be cosmetically undesirable as the new tissue formed during secondary intention healing may not match the color, texture, and thickness of the original and surrounding skin. Additionally, if the surgical defect was in an area with hair, secondary intention healing might not restore hair growth in that region.

Primary closure or side-to-side closure is a surgical technique where the edges of a wound or surgical defect are brought together and sutured, which allows the wound to heal by directly joining the skin or tissue. It is a common method for closing wounds when there is enough available healthy skin or tissue to do so. In this context, using the side-to-side closure method is not a feasible or practical option because the surgical defect is too large and there is not enough lax (loose or pliable) skin in the surrounding area to allow for the closure of this large defect. Attempting to close such a large wound in this manner would likely result in excessive tension on the surrounding skin, which could lead to complications like wound dehiscence or poor wound healing.

Skin grafts are pieces of skin, often taken from one part of the body which are transplanted to another area to repair a defect or wound. Grafts might alter or distort the natural anatomy of the area where they are placed. This can result in changes to the appearance or function of that region, which may not be desirable as the transplanted tissue might not match the color and texture of the surrounding skin, so this can lead to a noticeable and potentially cosmetically problematic difference in skin texture. Additionally, grafts may contract or shrink over time. This can lead to a tightening or pulling effect on the grafted area, which may result in functional or aesthetic problems. For example, contracture can limit joint mobility or cause discomfort. Moreover, in some cases, skin grafts may not fully integrate with the surrounding tissue, leading to a sunken or depressed appearance at the graft site. Therefore, skin grafts may not be the preferred choice for the reconstruction of surgical defects like ours.

Several techniques have been described to reconstruct defects like the one affecting our patient. Shea et al. reported the reconstruction of a similar left cheek defect with a rotational advancement or cervicofacial flap [6]. It is called “cervicofacial” because it encompasses both the cervical (neck) and facial regions and it is mostly used to close medium to large defects of the lateral cheek, particularly those greater than 3 cm in size. The flap receives its blood supply from perforators originating from different arteries, depending on whether it is anteriorly or posteriorly based. For anteriorly based flaps, perforators are from the facial and submental arteries, while posteriorly based flaps receive their blood supply from the superficial temporal artery and preauricular vessels. The cervicofacial flap has some advantages. Firstly, it is a well suited flap for covering substantial defects in the cheek area. One significant advantage is that a substantial portion of the resulting scar can be concealed along the hairline and the neck, making it less noticeable. Furthermore, the superolateral extension of the flap helps reduce tension on the lower eyelid, which can be important for both functional and aesthetic reasons. Moreover, the skin from the flap tends to provide an excellent color match to the surrounding cheek skin, which is important for achieving a natural appearance after the surgery. All these reasons make the cervicofacial flap a versatile and effective option for addressing large cheek defects, offering both functional and cosmetic benefits. Its versatility in terms of blood supply and positioning makes it a valuable tool in reconstructive surgery.

Another feasible reconstructive technique for such a large cheek defect is a rotational bilobed flap. Meissner reported a mento-buccal bilobed flap for the reconstruction of a larger defect located in the left lateral cheek [7]. The first flap is taken from the cheek below the defect. Importantly, it is designed not to cross the nasolabial fold, which is the crease running from the side of the nose to the corner of the mouth. This flap is positioned to address the primary defect. The second flap is obtained from the submental region below the chin. This flap is intended to address the second defect. The rotational bilobed flap is a versatile surgical technique used for closure in various areas of the face, and it can yield satisfactory cosmetic outcomes for defects of different sizes.

Free flaps are primarily recommended for the reconstruction of large partial or full-thickness cheek defects. They involve the transplantation of tissue from a distant donor site. When free flaps are used for cheek reconstruction, they are often obtained from donor sites such as the anterolateral thigh (ALT), radial forearm, or rectus abdominis. The choice of donor site depends on various factors, such as the patient’s overall health and the specific requirements of the reconstruction. Appropriate selection of patients undergoing free flap reconstruction is crucial to assess the suitability for the procedure. Patients with vascular comorbidities may be at greater risk of complications that could compromise the viability of the flap after transplantation. In our case, we did not consider this type of reconstruction as the patient had enough nearby tissue reservoir to perform the aforementioned reconstructive technique.

## 4. Conclusions

There are several techniques available for closing larger lateral–medial cheek defects, each with its own advantages and potential complications. The selection of the most appropriate method for closing a larger cheek defect is a complex decision that involves multiple factors, including functional and cosmetic considerations, as well as the specific characteristics of the defect. Surgeons should carefully evaluate each case to determine the best approach to achieve the desired functional and cosmetic results while optimizing wound healing. We have described a novel method for the reconstruction of larger cheek defects achieving optimal cosmetic and functional results.

## Data Availability

The data that support the findings of this study are available from the corresponding author upon reasonable request.
